# Caffeine Sources and Consumption among Saudi Adults Living with Diabetes and Its Potential Effect on HbA1c

**DOI:** 10.3390/nu13061960

**Published:** 2021-06-07

**Authors:** Salwa Ali Albar, Merfat Abdulrahman Almaghrabi, Rawabi Ahmed Bukhari, Rawan Hussein Alghanmi, Maha Ali Althaiban, Khaled A. Yaghmour

**Affiliations:** 1 Food and Nutrition Department, King Abdulaziz University, P.O. Box 80200, Jeddah 21589, Saudi Arabia; rawabi.bukhari@gmail.com (R.A.B.); ralghanmi0027@stu.kau.edu.sa (R.H.A.); mthaiban@kau.edu.sa (M.A.A.); 2 Family Medicine Department, Faculty of Medicine, King Abdulaziz University, P.O. Box 80200, Jeddah 21589, Saudi Arabia; Kyaghmour@kau.edu.sa

**Keywords:** coffee, caffeine consumption, type II diabetes, Saudi adults, HbA1c, cardiovascular risk factors

## Abstract

Information regarding the spread and effect of coffee and caffeine intake by individuals with type II diabetes remains unclear. This study aims to identify the amount and sources of habitual caffeine intake by individuals with type II diabetes and to investigate its association with other health outcomes, especially HbA1c. This is a cross-sectional survey involving 100 people medically defined as having type II diabetes comprising both genders, recruited from a care centre. All participants completed a caffeine semi-quantitative food frequency questionnaire (C-FFQ) to estimate their caffeine consumption, a two day 24-h recall, and a detailed questionnaire. The average caffeine intake was calculated from all sources and the differences in mean by gender were tested using a regression model (adjusted to important confounders). Regression models were used to verify the association between average caffeine intake on HbA1c and other health outcomes with adjustment for important confounders. A *p* value < 0.05 represented statistical significance. Arabic coffee (gahwa) and tea were the most common sources of caffeine among Saudi adults living with diabetes. Average caffeine intake for the whole sample was 194 ± 165 mg/day, which is 2.3 ± 2 mg/kg. There was an inverse association between caffeine intake and age: difference in mean −3.26 mg/year (95%CI: −5.34, −1.18; *p* = 0.003). Males had significantly higher consumption of caffeine compared to females: difference in mean 90.7 mg/day (95%CI: 13.8, 167.6; *p* = 0.021). No association was found between average caffeine intake and HbA1C or any other cardiovascular risk factors. This information can help public health practitioners and policy makers when assessing the risk of caffeine consumption among this vulnerable group.

## 1. Introduction

Diabetes is a major health issue that has reached alarming levels all over the world. The number of people affected by diabetes is expected to exceed 578 million by 2030 and 700 million by 2045 [1]. The highest prevalence of diabetes is anticipated to occur in the Middle East and North Africa as a result of rapid economic development, urbanization, and changes in lifestyle patterns in these regions [1]. Saudi Arabia ranks as the second highest in the Middle East and seventh in the world in terms of the rate of diabetes, according to the World Health Organization (WHO). It is estimated that around 7 million people in the population live with diabetes and almost 3 million are at the pre-diabetes stage [2].

People living with diabetes commonly experience other associated chronic conditions, resulting in serious complications. It is estimated that over 90% of diabetes patients are vulnerable to diseases such as kidney failure, heart attack, stroke, and lower limb amputation [3]. This places massive pressure on health care systems.

Globally, between 2000–2019 there was a 70% rise in deaths from diabetes, with an 80% rise in deaths among males. In the Eastern Mediterranean, the percentage has more than doubled and it now represents the greatest increase of all WHO regions [4]. Epidemiologic studies and randomized clinical trials show that type II diabetes is largely preventable through diet and lifestyle alterations [5,6].

Approximately 80% of the world’s population currently consumes caffeinated products every day; therefore, caffeine is the most widely consumed psychoactive component in the world. Caffeine is found mostly in beverages such as coffee, tea, energy drinks, and chocolates [7]. However, its intake varies depending on the type of beverages and also among different populations [7]. Caffeine is a central nervous system stimulant of the methylxanthine and plays an important role in increasing alertness, reducing fatigue, improving performance, and increasing mental functioning; it can also reduce blood glucose [1,8,9,10].

Many studies have shown that coffee consumption is more likely to have a beneficial rather than harmful effect on an individual’s health, although this depends on the amount of coffee consumed. It lowers the risk of all-cause mortality and cardiovascular mortality, as well as lowering the risk of incidence of cancer, metabolic issues including type II diabetes, and neurological conditions such as depression and Parkinson’s disease [11,12].

In Western populations, caffeinated and decaffeinated coffee consumption is associated with a reduced risk of type II diabetes [13]. The ingesting of caffeinated coffee has been found to be associated with a 4% lower risk of type II diabetes [14]. According to short-term studies in healthy adults, caffeine ingestion leads to impaired glucose tolerance and acute transient insulin resistance [15,16]. Various studies have confirmed that healthy, habitual coffee consumers are safe from the risk of diabetes compared to non-consumers. Coffee consumption has been correlated with a decreased occurrence of disabled glucose tolerance, insulin sensitivity, and hyperglycemia [17,18,19,20].

Some studies, however, have found no significant effect of coffee consumption on incidence of diabetes [21,22]. Others have found that there is a significant improvement in glucose control of HbA1c after stopping drinking caffeinated beverages over a three month follow-up [23]. In one experimental study it was found that caffeine had adverse effects on glucose metabolism, increasing average daytime glucose concentrations and exaggerating glucose levels after meals when habitual coffee drinkers with type II diabetes were monitored [24]. This suggests that daily consumption of caffeinated beverages can hinder clinical efforts to control glucose and might increase health complications [24]. On the other hand, caffeine and caffeinated coffee have been shown to increase blood pressure, and thus may pose a health threat to persons with cardiovascular disease [25,26].

In Saudi Arabia, the Arabian coffee ‘gahwa’ is a common hot drink that is boiled and served daily in all local social occasions and gatherings. Gahwa is served in small cups (around 30 mL each) and is usually consumed with dates and sweets [27]. Information regarding the spread of coffee and caffeinated products consumed by the Saudi population, particularly individuals with type II diabetes, remains unclear [28,29]. An exploration of the effect of habitual caffeine intake among adults with diabetes in the Saudi context is needed [30]. The prevalence of type II diabetes is high among the Saudi adults and considered a major public health problem. The Saudi population believe that they are heavy coffee drinkers [31] and evidence from trials among people with type II diabetes indicates a significant negative effect of caffeine ingestion (200–500 mg) on blood glucose control [32].

This study aims to identify the amount and sources of caffeine consumed by Saudi individuals with established type II diabetes and explore its association with other health outcomes, especially the level of glycated hemoglobin (HbA1c). This is in order to provide public health practitioners and dietitians with information on caffeine consumption among Saudi adults living with type II diabetes, to help them understand the situation and make suitable recommendations for patients.

## 2. Materials and Methods

### 2.1. Study Population

A cross-sectional study was undertaken of 100 patients medically defined with type II diabetes comprising both genders. Eighty participants were required to give 90% power to detect a 25% (191 ± 129 gm/day) [33] difference in the mean of caffeine consumption at a significant level of 0.05. Allowing for a 20% attrition rate, the aim was to recruit 96 participants.

They were randomly recruited from Jeddah Care Centre for Diabetics and Hypertension (this institution is considered the first and largest public institution in Jeddah and serves a large number of people from all areas) during the months of October 2019 to December 2019. We excluded all young patients (less than 20 years old), pregnant women, and those in the lactation stage. Medical information for the study was collected from patients’ medical records, while the questionnaire and nutritional information collection (two non-consecutive days of 24-h dietary recall followed by a caffeine food frequency questionnaire (FFQ)) were undertaken by trained nutritionists.

Data collection was performed twice: the first time was via a face-to-face interview and the second via telephone. A third telephone interview was carried out if there was any missing or unclear information. The study was conducted in accordance with the Declaration of Helsinki and the protocol was approved by the Ethics Committee of King Abdulaziz University, Unit of Biomedical Ethics (Ethic reference: 127-21). All participants provided written informed consent to participate in the study.

### 2.2. Assessment of Medical History, Anthropometric Measurement, and Lifestyle

All medical information regarding HbA1c, systolic and diastolic blood pressure, serum levels of total cholesterol, high density lipoprotein (HDL), low density lipoprotein (LDL), triglycerides, any history of disease in first-degree relatives, medication, weight, height, and waist circumference was collected from patients’ medical records. Only reports that had been updated one week before the interview were considered in this study to cover the same timeframe as the C-FFQ data. Information regarding patients’ lifestyles, such as smoking, physical activity, and dietary behaviour was collected through the face-to-face interview. BMI was calculated as weight in kilograms divided by the square of height in meters.

### 2.3. Estimating Caffeine Consumption

#### 2.3.1. 24-h Dietary Recall

Each participant was asked to complete two non-consecutive days of 24-h dietary recall to estimate their average energy intake (EI). In addition, booklets showing food portion size pictures were provided to participants in order to facilitate their dietary recall and gather accurate information. Information regarding food and beverage intake was collected using a multiple-pass approach of interview methodology [34]. Nutrient intake was calculated using computerised dietary analysis software. The program used calculated nutrient intake based on the US Department of Agriculture (USDA) food database.

#### 2.3.2. Caffeine Food Frequency Questionnaire

A semi-quantitative caffeine food frequency questionnaire (C-FFQ) was developed and validated previously by the authors (data under review). The C-FFQ includes common drinks and food items that are sources of caffeine and consumed by Saudi adults. The C-FFQ includes 6 main categories including energy drinks (10 items), soft drinks (11 items), tea (black tea and green tea; 8 items), coffee (caffeinated and decaffeinated coffee; 11 items), Arabic coffee (Arabic coffee and husk coffee), and chocolate and hot chocolate (12 items). The participants were asked if they consumed other drinks or chocolate items regularly that may contain caffeine, in order to add these to the FFQ. However, most of the added drinks were seasonal and did not contain caffeine, such as ‘sahlab’, which is a hot drink prepared from milk and corn flour. Only those food items that have a similar serving size and caffeine amount were grouped together, such as soft drinks (e.g., Pepsi, Diet Pepsi, and Coca Cola).

Participants reported on the frequency of consumption (ranging from ‘never or less than once per month’ to ‘6+ per day’), portion size (e.g., small, medium, or large, with a medium serving size being described as the usual serving size of a drink, such as ‘one cup or glass’), different types of caffeinated soft drinks (‘bottle or can’), and chocolate products (‘bar or packet’). The amount of caffeine in each drink or food item in the FFQ was identified using the USDA database [35].

For those drinks that were not available in the USDA (for example, information regarding Arabic coffee and Arabic coffee husks), other scientific published data were used to update the table [31,36,37]. To assess the total intake of caffeine in the FFQ, we added caffeine content for a specific amount and multiplied by a weight proportional to the frequency of its use [38]. The C-FFQ asks about beverages and foods that have been consumed over the last three months. The average daily total caffeine consumption from beverages and food sources was calculated and expressed as mg/day [38].

### 2.4. Statistical Analysis

Descriptive statistics means (SD) were used to describe the general characteristics, energy intake (EI), and macronutrients of the whole sample, after being stratified by gender. A Pearson Chi Square test was used to test the differences between the proportion of males and females in terms of general characteristics, smoking, and BMI classification. BMI status was classified based on the WHO [39], whereby less than 18.5 is underweight, 18.5 to less than 25 is normal, 25 to less than 30 is overweight, and 30 ≥ is obese. For continuous variables (EI) an independent two-sample *t*-test (a two-tailed test) was used.

Summary statistics for the mean (SD), and median, with the 25th and 75th percentile for caffeine intake (mg/day) as its source, were calculated and presented. The association between caffeine consumption and gender was investigated using a regression (model-1), with caffeine consumption (mg/day) as a dependent variable and gender as a predictor variable adjusted for confounders (age, BMI, smoking, and EI). A similar analysis has been done with the intake of caffeine/body weight (mg/kg) as a dependent variable.

Some sources of caffeine consumption (coffee, Arabic coffee, soft drinks, energy drinks, and chocolate) were log transformed when the assumption of normality was not met, as it yields similar variances and interpretable results after back-transforming the estimate [40]. The logged data were then tested and the results were back-transformed by anti-logging to return to the original scale. Thus, the back-transformed difference and 95%CI were presented as ratios of the geometric means (female to male ratio).

In addition, association between levels of HbA1c, cholesterol, triglyceride, and blood pressure for all participants as the dependent variables and total caffeine intake (100 mg/day) as an independent (predictor) variable with adjustment for BMI, age, gender, smoking, and EI were examined using regression (model-2) for the whole sample. A similar analysis was conducted after stratifying the sample by gender. When stratifying the sample by gender, association was done using a regression (model-3) adjusted for BMI, age, smoking, and EI. A statistical significance level of *p* < 0.05 was used. Analyses were carried out using Stata statistical software release 12 (Stata Corporation) [41].

## 3. Results

### 3.1. Sample Characteristics

Table 1 illustrates the sample characteristics as a whole and after stratification by gender. A total of 100 participants with type II diabetes were recruited from Jeddah Care Centre for Diabetics and Hypertension (CCDH). The average age of participants was 56 ± 14 years and 50% were male. Mean BMI was higher in females than males by 2.7 kg/m^2^; (95%CI: 0.19, 5.28) and this difference was statically significant at *p* = 0.035. A total of 36% of the participants mentioned that they were following a special diet, mainly a diabetes diet (83%). The average EI for the sample was 2392 kcal (610), with no difference between males and females in the proportion of energy intake from dietary fat.

In terms of participants’ general health, 74% of them were medically diagnosed as having a high cholesterol level, 60% had high blood pressure, and 28% had another disease (such as heart disease, kidney disease, or both). No differences were found in the proportion of these diseases by gender. In addition, there were no significant differences between males and females in the sample in terms of their duration of having diabetes or in terms of the type of treatments they used (Table 1).

A total of 17% of the participants were smokers and the number of smokers was significantly higher among males compared to females, at 24% male smokers to 10% female smokers, *p* < 0.001. Only 39% of the total participants practiced physical activity more than four times a week and there were no significant differences in the proportion by gender.

### 3.2. Source and Amount of Caffeine Consumption by Saudi Adults Living with Diabetes

The mean (SD) and median (IQR) of total caffeine consumption from the FFQ were calculated and are illustrated by source and gender in Table 2. The findings show that males consumed more caffeine than females and the difference was statistically significant, with the adjusted difference in mean 90.70 mg/day (95%CI: 13.8, 167.6; *p* = 0.021) for important confounders (BMI, smoking, kcal, age). Furthermore, there were significant differences in the mean caffeine consumption per body weight between males and females, with the adjusted difference in mean 1.29 mg/kg (95%CI: 2.27, 0.35; *p* = 0.011) (Table 2).

Arabic coffee (gahwa) and tea were the most common sources of caffeine among Saudi adults living with diabetes, followed by soft drinks, chocolate, and coffee (the number of consumers were 95, 95, 90, 56, and 52, respectively). Energy drinks ranked as the lowest source of caffeine in the study population, with only 12 consumers. Females consumed more caffeine from Arabic coffee and chocolate than males (ratio 1.5 (50%) and 1.9 (90%) respectively), but these differences were not significant. Overall, there were no significant differences between males and females in terms of average caffeine consumption by different sources (Table 2).

There was, however, an inverse association between total caffeine intake and age, changes in mean −3.26 mg/year (95%CI: −5.34, −1.18; *p* = 0.003), adjusted to BMI, smoking, and kcal confounders. Furthermore, there was an inverse association between caffeine intake from soft drinks and total chocolate and age, with significantly adjusted changes in mean−0.841 mg/year (95%CI: −1.42, −0.260; *p*= 0.005) and −0.183 mg/year; (95%CI: −0.35, −0.02; *p* = 0.028), respectively.

### 3.3. Caffeine Intake and Other Health Outcomes

Table 3 illustrates the association between caffeine intake and changes in the level of HbA1c, cholesterol, triglyceride, and blood pressure for all participants and after stratification of the sample by gender. We re-parameterised the clinical outcomes to indicate the change in them for an amount of caffeine intake equivalent to 100 mg (an approximately 8-ounce cup of coffee closer to 80 to 100 mg caffeine). There was no significant association found between the level of HbA1c or cholesterol, triglyceride, and blood pressure and caffeine intake (100 mg/day). Changes in the HbA1c mean/100 mg caffeine: 0.3 (95%CI: −0.3, 0.3; *p*= 0.866; r^2^ =0.0643). This suggests that 6.4% of the variance in the level of HbA1c can be explained by the consumption of caffeine among Saudi adults living with diabetes. This might be the reason for the insignificant results in the regression model-3. There were similar findings when adjusted to duration of diabetes and type of treatments.

## 4. Discussion

This paper has estimated caffeine intake mg/day and identified its sources among individuals with type II diabetes, exploring its association with other health outcomes. To our knowledge there is no information about caffeine consumption among the Saudi population in general and, more importantly, among this vulnerable group [27,42]. The current findings indicate that Arabic coffee (gahwa) and tea are the main sources of caffeine in drinks for Saudi adults living with diabetes. Other types of coffee, such as espresso, latte, cappuccino, etc., were consumed by about half of the participants, while energy drinks were ranked as the lowest source of caffeine among them. There was a positive association between gender and caffeine intake, with males consuming more caffeine than females. However, there was a negative association found between age and total caffeine intake, in particular in the case of certain caffeine sources such as soft drinks and total chocolate. The average daily caffeine consumption was found to be 194 ±165 mg/day and there was no association found between average caffeine intake and HbA1c or other health outcomes.

Two billion cups of coffee are estimated to be consumed every day globally [11]. Caffeine is considered to be the component responsible for the beneficial effects of coffee, but some studies also suggest the role of other compounds in health outcomes [43]. Caffeine is naturally found in cocoa beans, tea, kola nuts, and seeds, especially coffee seeds. Several studies have revealed that coffee is the primary source of caffeine. However, caffeine can be found in energy drinks, colas, chocolates, alcohol, and some medicines.

Coffee is the main source of caffeine among adults in most countries, with Finland and Norway at the top of the list, with averages of 9.6 and 7.2 kg of coffee consumed per capita per year. The U.S. ranks 22nd, at 3.1 kg per capita per year. A Canadian study found that coffee was the second most popular drink among Canadian adults after water. On the other hand, and similar to our findings, tea was found to be the main source of caffeine in Ireland (59%) and the UK (57%) [44]. Colas, energy drinks, and chocolate were found to be minor sources of caffeine among adults [44].

Although the Saudi population believe they are heavy coffee drinkers [33] they consume low amounts of caffeine. The data reveal that the mean total daily caffeine consumption among Saudis living with type II diabetes is below the caffeine dose level that is established by the European Food Safety Authority (EFSA), which is 400 mg/day for adults [45]. This is in line with other countries; for example, the average caffeine consumption in the UK is about 130 mg/day [46], and it is 191 ± 129 mg/day in Switzerland [33]. However, in Japan, they consume about 260 mg/day [47] and in the U.S. adults consume 233 mg/day, which is about the amount of caffeine in up to two cups of coffee [37]. Knowing the source of caffeine can explain why some nations and age groups have a higher consumption of caffeine than others. In Saudi Arabia, most of the adults living with diabetes consume tea, and Arabic coffee which has a different method of preparation and lower amounts of caffeine content compared to other types of coffee. Each cup of Arabian coffee contains only 4 mg of caffeine per 25 mL [31]. Based on the EFSA report, one of the main limitations and gaps in the available caffeine intake data is the limited information on all sources of caffeine variation by age group and gender, and in specific populations. Such differences do not allow for direct comparison between countries [45].

In most studies, age, sex, BMI (or obesity), and smoking are considered as potential confounders and are adjusted for potential confounding [16,22,48,49]. This is of importance, as in this study it was found that higher coffee consumption is associated with being male. Similarly, using National Health and Nutrition Examination Survey (NHANES) data covering *N* = 24,808 adults (≥19 years old) it can be seen that men have a significantly higher usual caffeine intake than women, at 240 ± 4 mg/day and 183 ± 3 mg/day, respectively [48].

Although caffeine consumption has been positively associated with age in several studies [22,49,50], a negative association with age was found in the current study and this may be due to the age range of the sample (the IQR 47–67). Findings from 903 elderly people in Spain, aged 65 years and above, showed that consumption of caffeinated coffee was negatively associated with age (≥75 years), RRR = 0.64 (0.43–0.94) [49].

Information regarding the health effects of caffeine consumption is controversial. There are some indications of its risks and various evidence of long-term health benefits among healthy adults who consume a moderate amount of caffeine based on the recommended level, such as prevention of the onset of diabetes [19,48,49,51,52].

In this study, caffeine consumption was found not to be significantly associated with the measure of chronic glucose control of HbA1c, even after stratification of the data by gender. This is in line with the findings of Watson et al. among adults with type I diabetes, where HbA1c, plasma lipid, and body weight measurements were not affected by caffeine status [53]. Evidence from a systematic review of 253 articles indicated that ingestion of caffeine (approximately 200–500 mg) significantly increases blood glucose concentration by 16–28% and decreases insulin sensitivity by 14–37% among individuals with type II diabetes [32].

In a pre and post-test design pilot study (*n* = 7) aimed at testing the effects of caffeine abstinence on HbA1c over three months among coffee drinkers with established type ll diabetes, it was found that coffee abstinence produced significant decreases in HbA1c and increases in 1,5-AG, both indicating improvement in chronic glucose control; no changes were found in the levels of fasting glucose or insulin [23]. This study did not report the amount of caffeine participants previously consumed, although this might explain the results.

One study highlighted that duration of diabetes by years correlated with the negative effect of caffeine on blood glucose [50]. However, no association between caffeine consumption and HbA1c was found in the current study, even when adjusted for duration of diabetes or type of treatment used (data not shown). This might be due to the caffeine consumption by our sample being within the safe limit recommended by EFSA for healthy people.

The consumption of dates with coffee is a common habit among Saudi people and this might affect hyperglycemic excursion after meals. A small study found that coffee did not impact capillary glycose levels and the glycemic indices of the Khalas data consumed with or without coffee among individual with type II diabetes was 53 ± 6 and 41.5 ± 5.4 respectively, at least in the short-term [54].

Furthermore, this study found no significant association between habitual caffeine intake and blood pressure (systolic and diastolic), and level of cholesterol. In a systematic review and meta-analysis of six prospective cohort studies it was suggested that habitual coffee consumption of >3 cups/d was not associated with an increased risk of hypertension compared with the lowest consumption <1 cup/d [51]. Mixed results were found in cohort studies on the association between habitual caffeine intake and long-term changes in blood pressure [45].

Generally, the panel in EFSA’s report indicated that scientific publications have identified almost no relationship or inverse relationship between caffeine intake and other adverse health effects [45]. The lack of association between habitual caffeine consumption and health outcome may be due to the dose of caffeine of 194 mg/day (2.3 mg/kg body weight) being less than the single dose of caffeine identified by EFSA. EFSA reported that single doses of caffeine from all sources up to 200 mg (about 3 mg/kg body weight) do not raise concerns for general adult health [45]. The estimated proportion of adults exceeding a daily intake of 400 mg ranged from 5.8% to 32.9% between different countries [45]. However, in this study only 10% of the sample exceeded 400 mg/day and they were mainly men.

There is no current guidance to suggest whether this recommendation should be reduced for people with diabetes. Generally, poorly controlled individuals with type II diabetes are encouraged to reduce their daily consumption of caffeine from 400 to 200 mg/day [32,52]. This is because it is proposed that caffeine impacts blood glucose concentrations in individuals with existing diabetes, by inhibiting glucose uptake into muscle cells, even in the presence of insulin [55]. Others suggest that caffeine may elevate epinephrine which may induce insulin resistance by the impairment of glucose uptake in the peripheral tissue [56].

The present study has some limitations. It is a cross-sectional survey and the nature of such a study means that determination of the direction of association is prevented. Thus, to lower the risk of an inverse causality and recall bias, human intervention studies and prospective cohort studies have been preferred over case control and cross-sectional studies. Larger-scale, randomised, controlled trials over a longer period and using a larger sample size are needed to identify the effects of doses of habitual caffeine consumption from different sources on HbA1c and other health outcomes [32]. In addition, this survey does not provide information about fasting blood sugar or caffeine obtained from non-dietary sources such as food supplements or medicines.

In this study, habitual caffeine consumption from different sources was considered, with an adequate control for confounding variables. To our knowledge this is the first study conducted among adults living with diabetes in Saudi Arabia investigating habitual caffeine intake from different sources; therefore, it can be considered an important and complex first step toward understanding the effects of caffeine on health, especially among adults with established diabetes. Most of the available studies focus on the effect of caffeine as a risk factor of diabetes among healthy adults.

## 5. Conclusions

In conclusion, the current findings suggest that Arabic coffee and tea are the main sources of caffeine among Saudi individuals with type II diabetes. Average consumption of caffeine among Saudi adults living with diabetes is less than the single dose of caffeine identified by the EFSA for healthy adults. Caffeine intake of the study sample was not associated with any significant changes in HbA1c or other health outcomes. Understanding variations between genders in terms of caffeine consumption and its sources is critical and the first step for all public health practitioners, dietitians, and policy makers when assessing the risk of caffeine consumption among this vulnerable group. Further research is needed to expand the current evidence and determine the effects of timing and dose of daily habitual caffeine consumption among free living adults with diabetes.

## Figures and Tables

**Table 1:** General characteristics of study participants by gender.

General Characteristics	All Participants (*n* = 100)		Females (*n* = 50)		Males (*n* = 50)		
	Mean	SD	Mean	SD	Mean	SD	*p*-Value
Age * (years)	56.2	14.0	57.1	14.4	55.3	13.7	0.650
Height * (cm)	161.9	8.1	157.4	7.0	166.5	6.3	0.001
Weight * (kg)	89.2	14.5	88.5	20.8	89.8	15.9	0.708
BMI * (kg/m^2^)	33.7	6.5	35.0	7.1	32.3	5.7	0.035
**BMI category † (n, %)**							
Normal weight	6	6%	3	6%	3	6%	
Overweight	26	26%	10	20%	16	32%	0.038
Obese	68	68%	37	74%	31	62%	
Waist circumference * (cm)	98	15	99	17	97	13	0.444
**Following special Diet † (yes, n, %)**	36	36%	18	36%	18	36%	1.000
Diabetes Diet (self-assessment of balanced diet)	30	83%	14	78%	16	89%	0.212
Diabetes Diet and other diet e.g., low fat diet or low salt diet or for losing weight	6	17%	4	22%	2	11%	
Duration of having diabetes (year)	14	10	13	8	15	11	0.305
**Diabetes treatment † (n, %)**							
Oral antidiabetic drugs only	37	37%	21	42%	16	32%	
Insulin	22	22%	9	18%	13	26%	0.490
Oral antidiabetic drugs and Insulin	41	41%	20	40%	21	42%	
Dietary characteristics *							
Total energy (Kcal)	2392	610	2194	614	2589	544	0.001
Protein (g)	102	46	80	34	123	46	0.001
Fat (g)	77	34	70	34	83	34	0.053
Energy from fat (Kcal)	708	349	746	387	746	305	0.273
Carbohydrate (g)	318	95	303	106	333	81	0.121
**Having chronic disease † (n, %)**							
Blood pressure (yes)	60	60%	33	66%	27	54%	0.221
Cholesterol (yes)	74	74%	38	76%	36	72%	0.648
Other disease (yes)	28	28%	16	32%	12	24%	0.373
**Smoking † (n, %)**							
Yes	17	17%	5	10%	12	24%	0.001
I have given up	23	23%	1	2%	22	44%	
**Physical activity † (n, %)**							
Never	28	28%	17	34%	11	22%	0.119
Once a week	21	21%	14	28%	7	14%	
Twice a week	12	12%	5	10%	7	14%	
More than four times week	39	39%	14	28%	25	50%	

* Differences between genders were assessed by using an independent sample *t*-test. † Differences between the proportion of males and females in the categorical variables were assessed by using a Chi2 test.

**Table 2:** Mean (SD) and median (IQR) of total caffeine intake (mg/day) by source and gender, and the tested mean differences between genders.

Source of Caffeine	All Participants			Females			Males			Adjusted Difference in Means (95%CI) *	
	N	Mean (SD)	Median (IQR)	N	Mean (SD)	Median (IQR)	N	Mean (SD)	Median (IQR)		*p*-Value
Caffeine-FFQ (mg/day)	100	194 (165)	151 (81, 233)	50	127 (107)	99 (52, 181)	50	261 (185.0)	218(121, 345)	90.70 (13.8, 167.6)	0.021
Caffeine/body weight (mg/kg)		2.3 (2.0)	1.8 (0.9, 2.8)	50	1.5 (1.4)	1.2 (0.7, 1.9)	50	3.0 (2.4)	2.3 (1.5, 3.4)	1.29 (2.27, 0.35)	0.011
Tea	95	116(108)	76 (44, 180)	46	79 (75)	72 (31, 103)	49	150 (123)	110(72, 187)	31.51(21.77,84.80)	0.243
										ratio of the geometric means (95%CI) †	*p*-value
Coffee	52	94 (128)	56 (10, 102)	22	66 (77)	47 (9, 94)	30	114 (154)	56 (18, 130)	0.53 (0.22, 1.24)	0.139
Arabic coffee (Gahwa)	95	8 (12)	3 (1, 8)	48	9 (12)	4 (2, 9)	47	7 (12)	2 (1, 8)	1.5 (0.67, 3.20)	0.338
Energy drink	12	31(45)	8 (5, 52)	5	29 (47)	8 (7, 11)	7	33.5 (34)	8 (3, 89)	0.89 (0.01, 61.9)	0.947
Soft drink	56	19 (31)	8 (5, 13)	28	14.5(20)	8 (5, 13)	28	24 (39)	8 (5, 19)	0.74 (0.36, 1.5)	0.406
Chocolate	90	6 (11)	2.3 (1, 5)	45	6 (12)	3 (1, 5)	45	6 (11)	2 (1, 5)	1.9 (0.89, 4.1)	0.654

*N* = number of consumers. * Differences between gender in mean caffeine consumption by sources were assessed by using regression model-1 with adjusting for BMI, age, smoking, and total energy intake (EI); † Transformed data, the difference was assessed using regression model-1 and after back-transformation the difference was the ratio of the sample geometric mean (female to male ratio).

**Table 3:** Association between caffeine intake (100 mg/day) and HbA1C, cholesterol, triglyceride, and blood pressure for all participants and clustering by gender.

Health Outcome	All Participants ^†^				Female ^‡^				Male ^‡^			
	N	Mean (SD)	Change in Health Outcome/100 mg Caffeine * (95%CI)	*p* Value	N	Mean (SD)	Change in Health Outcome/100 mg Caffeine (95%CI)	*p* Value	N	Mean (95%CI)	Change in Health Outcome/100 mg Caffeine (95%CI)	*p* Value
HbA1c	100	8.5 (2.1)	0.3 (−0.3, 0.3)	0.866	50	8.5 (2.3)	0.3 (−0.4, 1.0)	0.348	50	8.6 (2.01)	−0.1 (−0.4, 0.3)	0.661
Cholesterol (mmol/L)	85	172.6 (53.0)	3.4 (−4.7, 11.7)	0.400	40	172.5 (58.2)	4.3 (−16.2, 24.8)	0.672	45	172.8 (48.66)	4.1(−4.8, 13.1)	0.358
HDL (mg/dL)	81	47.7 (21.8)	1.5 (−1.97, 5.02)	0.389	39	50.6 (24.3)	3.2 (−5.6, 11.9)	0.464	42	44.9 (19.03)	1.1 (−2.5, 4.9)	0.519
LDL (mg/dL)	75	100.9 (45.6)	0.72 (−6.4, 8.1)	0.844	34	102.3 (48.3)	9.9 (−7.5, 27.4)	0.252	41	99.7(43.80)	−1.6 (−10.1, 6.9)	0.706
Triglyceride (mg/dL)	87	137.5 (100.2)	2.0 (−13.1, 17.1)	0.791	42	141.9 (116.1)	−6.7 (−45.4, 31.8)	0.724	45	133.4 (84.01)	3.5 (−11.9, 19.1)	0.645
Blood pressure (mmHg)												
Diastolic	96	71.0 (10.6)	−1.1 (−2.4, 0.33)	0.135	48	68.4 (9.5)	−0.9 (−3.8, 2.0)	0.529	48	73.5 (11.06)	−1.1 (−2.7, 0.6)	0.194
Systolic	96	140.8(21.9)	−0.7 (−3.9, 2.4)	0.646	48	139.7 (20.5)	−1.5 (−7.8, 4.7)	0.624	48	141.9 (23.33)	−0.4 (−4.3, 3.64)	0.855

*N* = number of participants’ complete information; * approximately 8-ounce cup of coffee closer to 80 to 100 mg caffeine. † The associations were assessed by using regression model-2 with adjusting for BMI, age, gender, smoking, and EI. ‡ The associations were assessed by using regression model-3 with adjusting for BMI, age, smoking, and EI.

## Data Availability

The data presented in this study are available in the King Abdulaziz University or upon request from the corresponding author.
